# Maternal risk factors for the VACTERL association: A EUROCAT case–control study

**DOI:** 10.1002/bdr2.1686

**Published:** 2020-04-22

**Authors:** Romy van de Putte, Iris A.L.M. van Rooij, Cynthia P. Haanappel, Carlo L.M. Marcelis, Han G. Brunner, Marie‐Claude Addor, Clara Cavero‐Carbonell, Carlos M. Dias, Elizabeth S. Draper, Larraitz Etxebarriarteun, Miriam Gatt, Babak Khoshnood, Agnieszka Kinsner‐Ovaskainen, Kari Klungsoyr, Jenny J. Kurinczuk, Anna Latos‐Bielenska, Karen Luyt, Mary T. O'Mahony, Nicola Miller, Carmel Mullaney, Vera Nelen, Amanda J. Neville, Isabelle Perthus, Anna Pierini, Hanitra Randrianaivo, Judith Rankin, Anke Rissmann, Florence Rouget, Bruno Schaub, David Tucker, Diana Wellesley, Awi Wiesel, Natalya Zymak‐Zakutnia, Maria Loane, Ingeborg Barisic, Hermien E.K. de Walle, Jorieke E.H. Bergman, Nel Roeleveld

**Affiliations:** ^1^ Department for Health Evidence, Radboud Institute for Health Sciences Radboud university medical center (Radboudumc) Nijmegen The Netherlands; ^2^ Paediatric Surgery Radboudumc Amalia Children's Hospital Nijmegen The Netherlands; ^3^ Department of Human Genetics Nijmegen The Netherlands; ^4^ Department of Clinical Genetics and School for Oncology & Developmental Biology (GROW) Maastricht University Medical Center Maastricht The Netherlands; ^5^ Department of Woman‐Mother‐Child University Medical Center CHUV Lausanne Switzerland; ^6^ Rare Diseases Research Unit Foundation for the Promotion of Health and Biomedical Research in the Valencian Region Valencia Spain; ^7^ Epidemiology Department National Institute of Health Doutor Ricardo Jorge Lisbon Portugal; ^8^ Department of Health Sciences University of Leicester Leicester UK; ^9^ Department of Health, Public Health Service Basque Government Basque Country Vitoria‐Gasteiz Spain; ^10^ Malta Congenital Anomalies Register Directorate for Health Information and Research Pietà Malta; ^11^ INSERM UMR 1153, Obstetrical, Perinatal and Pediatric Epidemiology Research Team (EPOPé), Center of Research in Epidemiology and Statistics Sorbonne Paris Cité (CRESS), DHU Risks in Pregnancy Paris Descartes University Paris France; ^12^ European Commission, Joint Research Centre (JRC) Ispra Italy; ^13^ Department of Global Public Health and Primary Care University of Bergen Bergen Norway; ^14^ Division for Mental and Physical Health Norwegian Institute of Public Health Bergen Norway; ^15^ National Perinatal Epidemiology Unit, Nuffield Department of Population Health University of Oxford Oxford UK; ^16^ Department of Medical Genetics University of Medical Sciences Poznań Poland; ^17^ South West Congenital Anomaly Register (SWCAR), Bristol Medical School University of Bristol Bristol UK; ^18^ Department of Public Health Health Service Executive – South Cork Ireland; ^19^ National Congenital Anomaly and Rare Disease Registration Service Public Health England Newcastle upon Tyne UK; ^20^ Department of Public Health Health Service Executive – South East Kilkenny Ireland; ^21^ Provinciaal Instituut voor Hygiene (PIH) Antwerp Belgium; ^22^ Registro IMER ‐ IMER Registry (Emilia Romagna Registry of Birth Defects), Center for Clinical and Epidemiological Research University of Ferrara, Azienda Ospedaliero‐Universitaria di Ferrara Ferrara Italy; ^23^ Auvergne registry of congenital anomalies (CEMC‐Auvergne), Department of clinical genetics, Centre de Référence des Maladies Rares University Hospital of Clermont‐Ferrand Clermont‐Ferrand France; ^24^ Tuscany Registry of Congenital Defects (RTDC) Institute of Clinical Physiology ‐ National Research Council / Fondazione Toscana Gabriele Monasterio Pisa Italy; ^25^ Register of congenital malformations of Reunion Island CHU Réunion St Pierre France; ^26^ Institute of Health & Society Newcastle University Newcastle UK; ^27^ Malformation Monitoring Centre Saxony‐Anhalt Medical Faculty Otto‐von‐Guericke University Magdeburg Germany; ^28^ Brittany Registry of congenital anomalies CHU Rennes, University Rennes, Inserm, EHESP, Irset (Institut de recherche en santé, environnement et travail) Rennes France; ^29^ French West Indies Registry, Registre des Malformations des Antilles (REMALAN), Maison de la Femme de la Mère et de l'Enfant University Hospital of Martinique Fort‐de‐France France; ^30^ CARIS, Public Health Wales Singleton Hospital Swansea UK; ^31^ Wessex Clinical Genetics Department Princess Anne Hospital Southampton UK; ^32^ Department of Pediatrics, Birth Registry Mainz Model University Medical Center of Mainz Mainz Germany; ^33^ OMNI‐Net Ukraine Birth Defects Program and Khmelnytsky City Children's Hospital Khmelnytsky Ukraine; ^34^ Centre for Maternal, Fetal and lnfant Research, lnstitute of Nursing and Health Research Ulster University Belfast UK; ^35^ Centre of Excellence for Reproductive and Regenerative Medicine, Children's Hospital Zagreb Medical School University of Zagreb Zagreb Croatia; ^36^ Department of Genetics, EUROCAT Northern Netherlands University of Groningen, University Medical Center Groningen Groningen The Netherlands

**Keywords:** etiology, assisted reproductive techniques, maternal factors, pregestational diabetes, respiratory disorders

## Abstract

**Background:**

The VACTERL association (VACTERL) is the nonrandom occurrence of at least three of these congenital anomalies: vertebral, anal, cardiac, tracheoesophageal, renal, and limb anomalies. Despite suggestions for involvement of several genes and nongenetic risk factors from small studies, the etiology of VACTERL remains largely unknown.

**Objective:**

To identify maternal risk factors for VACTERL in offspring in a large European study.

**Methods:**

A case–control study was performed using data from 28 EUROCAT registries over the period 1997–2015 with case and control ascertainment through hospital records, birth and death certificates, questionnaires, and/or *postmortem* examinations. Cases were diagnosed with VACTERL, while controls had a genetic syndrome and/or chromosomal abnormality. Data collected included type of birth defect and maternal characteristics, such as age, use of assisted reproductive techniques (ART), and chronic illnesses. Multivariable logistic regression analyses were performed to estimate confounder adjusted odds ratios (aOR) with 95% confidence intervals (95% CI).

**Results:**

The study population consisted of 329 VACTERL cases and 49,724 controls with recognized syndromes or chromosomal abnormality. For couples who conceived through ART, we found an increased risk of VACTERL (aOR 2.3 [95% CI 1.3, 3.9]) in offspring. Pregestational diabetes (aOR 3.1 [95% CI 1.1, 8.6]) and chronic lower obstructive pulmonary diseases (aOR 3.9 [95% CI 2.2, 6.7]) also increased the risk of having a child with VACTERL. Twin pregnancies were not associated with VACTERL (aOR 0.6 [95% CI 0.3, 1.4]).

**Conclusion:**

We identified several maternal risk factors for VACTERL in offspring befitting a multifactorial etiology.

## 1 INTRODUCTION

The VACTERL association (VACTERL) is a very serious condition that includes at least three of the following congenital anomalies: vertebral, anorectal, cardiac, tracheoesophageal fistula with or without esophageal atresia (EA/TEF), renal, and limb anomalies (Solomon, 2011). As no major genetic risk factors have been identified yet and no genetic test is available, VACTERL is diagnosed by excluding overlapping conditions that have multiple features in common with VACTERL (Solomon, 2011). In most patients, VACTERL occurs sporadically, but familial inheritance and increased prevalence rates of component features in first‐degree relatives are observed (Hilger et al., 2012; Reutter & Ludwig, 2013; Solomon, Pineda‐Alvarez, Raam, & Cummings, 2010). According to the data published by EUROCAT, the European network for the surveillance of congenital anomalies, the prevalence of VACTERL was 0.4 in 10.000 births in 2012–2017 (EUROCAT, 2019b). The majority of VACTERL patients who survive the immediate postnatal period undergo a series of complex surgical procedures to restore normal organ function. The quality of life is often reduced, as the patients frequently suffer from sequelae, such as back pain resulting from vertebral anomalies, incontinence or severe constipation as a result of an anorectal malformation (ARM), dysphagia or gastroesophageal reflux as a consequence of tracheoesophageal fistula (TEF), and urinary tract infections due to renal anomalies (Raam, Pineda‐Alvarez, Hadley, & Solomon, 2011; Wheeler & Weaver, 2005).

Until now, no major risk factors are known to be involved in the etiology of VACTERL. The phenotypic heterogeneity among VACTERL patients is large (van de Putte, van Rooij, et al., 2019), which makes etiologic research into VACTERL difficult, as strong etiological heterogeneity is likely (Solomon, 2018). There is evidence that a proportion of patients have a genetic basis, but other than the suggestion of certain candidate genes based on animal studies or case reports, no major genetic risk factors have been identified (Aguinaga, Zenteno, Perez‐Cano, & Moran, 2010; Garcia‐Barcelo et al., 2008; Winberg et al., 2014). Although it is certainly possible that genetic risk factors are involved in the etiology of VACTERL, maternal risk factors are hypothesized to play a role as well (Solomon, 2018). For example, maternal diabetes and assisted reproductive techniques (ART) were identified as possible maternal risk factors. However, the evidence for involvement of these factors in the etiology of VACTERL is not strong, as it is mainly based on case reports (Castori, Rinaldi, Capocaccia, Roggini, & Grammatico, 2008; Kanasugi et al., 2013; Sunagawa et al., 2007) or studies with very small sample sizes (Czeizel & Ludanyi, 1985; van Rooij et al., 2010; Zwink et al., 2012).

In this study, we included the largest group of VACTERL cases thus far described with the aim of identifying maternal risk factors in the etiology of VACTERL in offspring.

## 2 METHODS

### 2.1 EUROCAT data collection

**FIGURE 1:**
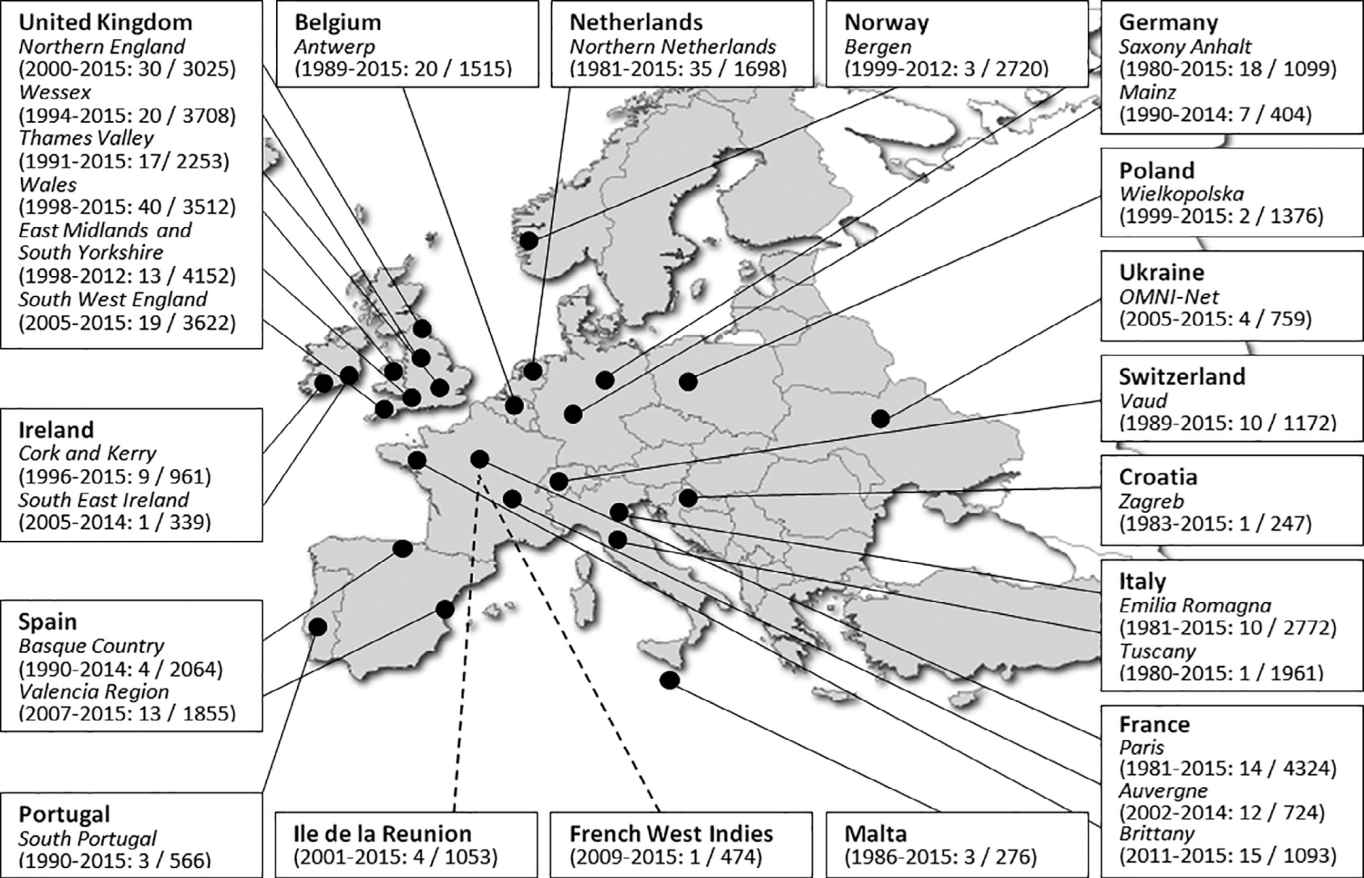
Map of the 28 participating EUROCAT registries with birth years covered and the total number of cases and controls that were included in this study

Standardized data from cases and controls were obtained from the central database of JRC‐EUROCAT, the European network of population‐based registries of congenital anomalies ("EUROCAT, 2019a). Registries report cases annually to the Central Registry operated at European Commission's the Joint Research Centre (JRC) in Ispra, Italy (Kinsner‐Ovaskainen et al., 2018; Tucker et al., 2018). Hospital records, birth and death certificates, maternal questionnaires, and *postmortem* examinations are all used to ascertain the data in individual registries. In this study, we used data from 28 full member EUROCAT registries from 15 European countries for the period between 1997 and 2015 (Figure 1). The International Classification of Diseases (ICD) version 9 or 10 and the British Pediatric Association (BPA) one digit extension were used to code all congenital anomalies, syndromes, chromosomal abnormalities, and maternal (chronic) illnesses. Data extraction was completed for all registered cases by the JRC‐EUROCAT Central Registry in May 2018. All 28 participating registries gave consent for their data to be extracted for this study.

### 2.2 Case and control selection

The initial study population comprised 441 cases with the VACTERL association and 50,165 controls with a genetic syndrome and/or chromosomal abnormality. Cases were selected based on ICD‐9‐BPA codes 759895 and 75989, ICD‐10‐BPA code Q8726, or OMIM/McKusick codes 192350, 314390, and 276950. Cases with an ICD‐9‐BPA code 75989 were only selected when VATER/VACTERL was specified in the text, as this ICD‐BPA code is not specific for VATER/VACTERL. We included live births, fetal deaths (miscarriages or stillbirths from 20 weeks gestation), and terminations of pregnancy for fetal anomaly following prenatal diagnosis at any gestational age (TOPFA). We excluded fetuses or infants with a diagnosis of VACTERL with hydrocephalus (VACTERL‐H), as this is considered a separate condition with a suggested autosomal recessive or X‐linked inheritance (OMIM %276950) (*n* = 11). Cases with a syndrome that explains their phenotype, such as Fanconi anemia, caudal regression syndrome, Goldenhar syndrome, sirenomelia, and the 22q11 deletion syndrome were also excluded (*n* = 11).

The remaining 419 VACTERL cases were subdivided into the three mutually exclusive VACTERL subtypes introduced earlier: STRICT‐VACTERL, VACTERL‐LIKE, and VACTERL‐PLUS (van de Putte, van Rooij, et al., 2019). The STRICT‐VACTERL subtype contains cases with ≥3 major VACTERL features. In the VACTERL‐LIKE subtype, we included cases with <3 major VACTERL features, but with additional minor VACTERL features adding up to ≥3 major and minor VACTERL features combined. The VACTERL‐PLUS subtype includes all cases that fulfill either the STRICT‐VACTERL or the VACTERL‐LIKE subtype criteria, but have additional major congenital anomalies outside the VACTERL spectrum. In addition, we introduced the NO‐VACTERL subgroup, containing patients originally diagnosed with VACTERL but actually not complying with the diagnostic criteria (van de Putte, van Rooij, et al., 2019). The NO‐VACTERL cases (*n* = 80) and cases for which the subtype was unknown due to missing information on the individual anomalies (*n* = 10) were excluded from further analyses. For some specific analyses, the numbers of cases and controls were lower because some registries do not collect information on all maternal risk factors in a systematic manner.

The control group included fetuses or infants with a genetic syndrome or chromosomal abnormality, consisting of live births, fetal deaths, and TOPFAs. We hypothesized that these controls have a different etiology from VACTERL cases, as genetic defects are minimally influenced by maternal factors. Controls were selected based on ICD‐9‐BPA codes: 75581, 75601, 75604, 7580–7583, 7585–7589, 7598, and 27910; and ICD‐10‐BPA codes: Q4471, Q6190, Q7484, Q751, Q754, Q7581, Q87, Q90‐Q93, Q96‐Q99, and D821. Frequently occurring syndromes among the controls were trisomy 21 (46%), trisomy 18 (11%), Turner syndrome (5%), and trisomy 13 (4%).

### 2.3 Exposure and outcomes

We included the following fetal/infant characteristics from the EUROCAT registries: sex, birth year, birth type (live birth, fetal deaths, TOPFA), survival (>1week of age), birthweight (in grams), and gestational age (in completed weeks). In addition, we included the following maternal characteristics: age at birth (in years), multiple pregnancy (vs. singleton pregnancy), the use of ART, including in vitro fertilization (IVF), intracytoplasmatic sperm injection (ICSI), gamete intrafallopian transfer (GIFT), oocyte donation, artificial insemination, or induced ovulation, chronic illnesses with onset before pregnancy, and drugs taken during the first trimester of pregnancy (from first day of last menstrual period up to 12 weeks of gestation). Parity and/or gravidity were not included as potential risk factors in this study, as information on parity and gravidity is not registered in a standardized way across the EUROCAT registries.

Maternal age at birth was divided into <20, 20–34, and ≥35 years. For ART, we made a distinction based on the invasiveness of the treatment: IVF, ICSI, GIFT, and egg donation were considered invasive ARTs, whereas artificial insemination and ovulation induction were considered noninvasive. Maternal illnesses included pregestational diabetes (ICD‐10 codes E10‐E14, P701, O240, and O241; ICD‐9 codes 2500–2509, 7750, and 6480), chronic lower obstructive pulmonary diseases (CLOPD) (ICD‐10 codes J40‐J47; ICD‐9 codes 490–496), and epilepsy (ICD‐10 codes: G400‐G409; ICD‐9 codes 3450–3459).

### 2.4 Statistical analysis

The statistical package IBM SPSS Statistics 25.0 (SPSS Inc., Chicago, IL, USA) was used for the analysis. Using logistic regression, we estimated crude odds ratios (ORs) with 95% confidence intervals (95%CIs) for several potential risk factors for VACTERL, namely multiple pregnancy, ART, pregestational diabetes, CLOPD, and epilepsy. In the multivariable analyses, the reporting registry was selected as a confounder a priori and was included in all models. In addition, birth year, birth type, and maternal age at birth were included as potential confounders, as well as the potential risk factors multiple pregnancy, ART, pregestational diabetes, and CLOPD that were not the primary factor of interest in the specific analysis. All potential confounders were initially included in the full model, from which they were subsequently excluded when the OR did not change more than 10% upon removal.

#### 2.4.1 Missing data

Registries were excluded from the analyses of specific risk factors when they had >50% missing data for that factor. As a result, the analyses regarding multiple pregnancy were based on all 28 registries, the analyses regarding ART on 16 registries, the analyses regarding pregestational diabetes on 15 registries, and the analyses regarding CLOPD and epilepsy on 14 registries. Within those datasets for analysis, the percentages of missing data varied from 0 to 23.8% for different variables. Therefore, we performed all analyses on the determinants of interest with multiple imputed data (50×) based on all maternal and child characteristics and potential risk factors, as displayed in Tables 2 and 3 using SPSS for imputation in addition to complete case analyses.

#### 2.4.2 Sensitivity analyses

Several sensitivity analyses were performed: (a) by using multiple imputation (50×) to deal with the remaining missing data after exclusion of registries with >50% missing data for specific analyses; (b) by inclusion of the NO‐VACTERL cases and cases with unknown subtype in the case population; (c) by inclusion of additional mothers with chronic illnesses based on information on drug use in the first trimester (ATC codes: A10 for drugs used in diabetes, R03 for drugs used for obstructive airway diseases, and N03 for antiepileptics); (d) by random exclusion of a proportion of the controls with TOPFA to achieve similar proportions of TOPFAs in the control group and the case group; (e) by assuming that cases and controls with missing data on any of the potential risk factors were not exposed to that factor; (f) by exclusion of controls with imprinting disorders (Beckwith‐Wiedemann syndrome, Angelman syndrome, Silver‐Russell syndrome, and Prader‐Willi syndrome) in the analyses regarding ART and controls with caudal regression syndrome in the analyses for gestational diabetes because of the known associations of ART with imprinting disorders (Manipalviratn, DeCherney, & Segars, 2009) and pregestational diabetes with caudal regression syndrome (Garne et al., 2012).

### 2.5 Ethics approval

As we used anonymous data obtained from registries with appropriate ethical approval, specific ethical committee approval was not required for this study.

## 3 RESULTS

The 329 VACTERL cases were divided into 173 cases (52%) belonging to the STRICT‐VACTERL subtype, 71 cases (22%) belonging to the VACTERL‐LIKE subtype, and 85 cases (26%) belonging to the VACTERL‐PLUS subtype. Table 1 shows the percentages of the major VACTERL component features as specified previously (van de Putte, van Rooij, et al., 2019) among the cases in this study. Further characteristics of our study population are described in Table 2. The majority of the cases were males (67%), whereas the proportions of males and females in the control population were equal. The distribution of birth years was quite similar for the case and control group. The majority of the cases were live born (76%), whereas this was 47% among the controls. On the other hand, the percentage of TOPFA pregnancies was 49% among the controls, and 22% in the cases. The survival of live born children beyond the first week postpartum was quite similar for cases and controls (88 vs. 96%), while low birthweight and preterm birth were observed approximately twice as often among the cases compared to controls (52 vs. 28% and 45 vs. 22%). Case mothers were younger than the mothers of controls with a genetic syndrome or chromosomal abnormality. Among the cases, 20% had a mother aged ≥35 years at birth, in comparison to 48% of the controls. Due to the nature of our control population, the higher percentage of TOPFA pregnancies and the higher maternal age were expected.

**TABLE 1:** The proportions of major VACTERL component features present in the 329 VACTERL cases included

	*N*	%
Vertebral anomalies	109	33.1
Anal anomalies	199	60.5
Cardiac anomalies	195	59.3
Tracheo‐esophageal anomalies	200	60.8
Renal anomalies	164	49.8
Limb anomalies	81	24.6

*Note*: See van de Putte, van Rooij, et al. 2019 for the specification of the congenital anomalies that belong to the major VACTERL component features.

**TABLE 2:** Fetal/infant and maternal characteristics of VACTERL cases and controls

	Missing data	Total number cases/controls	Cases *N* (%)	Controls *N* (%)
Sex	7.2%	326/46,099		
Male			217 (66.6)	23,136 (50.2)
Female			109 (33.4)	22,862 (49.6)
Indeterminate			—	101 (0.2)
Year of birth or TOPFA	0%	329/49,724		
1997–2000			39 (11.9)	6,273 (12.6)
2001–2005			71 (21.6)	11,128 (22.4)
2006–2010			113 (34.3)	16,557 (33.3)
2011–2015			106 (32.2)	15,766 (31.7)
Birth type	0.1%	329/49,697		
Live birth			250 (76.0)	23,560 (47.4)
Stillbirth			5 (1.5)	1,668 (3.4)
TOPFA			74 (22.5)	24,469 (49.2)
Survival (>1 week postpartum)^a^	7.2%	246/21,860	217 (88.2)	20,892 (95.6)
Low birthweight (<2,500 g)^a^	11.0%	245/20,947	127 (51.8)	5,865 (28.0)
Preterm birth (<37 weeks)^a^	5.0%	248/22,360	111 (44.8)	4,885 (21.8)
Maternal age at birth	2.0%	327/48,727		
<20 years			16 (4.9)	1,198 (2.5)
20–34 years			246 (75.2)	24,354 (50.0)
≥35 years			65 (19.9)	23,175 (47.5)

*Notes*: The numbers in this table are based on all 28 individual registries.

Abbreviation: TOPFA, terminations of pregnancy for fetal anomaly following prenatal diagnosis.

a Only calculated for live births.

In Table 3, the associations between the potential maternal risk factors and VACTERL in offspring are shown. Birth type, maternal age at birth, and ART proved to be true confounders in some multivariable analyses. Couples who conceived through ART had an increased risk of having a child with VACTERL (aOR 2.3 [95% CI 1.3, 3.9]), with a slight difference in risk between invasive and noninvasive techniques, such as artificial insemination or hormonal treatment (aOR 1.9 (95% CI 0.9, 4.0) and aOR 2.8 (95% CI 1.3, 6.1), respectively). We observed a more than threefold increased risk of VACTERL in offspring of mothers with pregestational diabetes (aOR 3.1 [95% CI 1.1, 8.6]) or CLOPD (aOR 3.9 [95% CI 2.2, 6.7]). Cases seemed more likely to be delivered from a multiple pregnancy (e.g., twin or triplet) compared to controls, but adjustment for birth type and ART attenuated the aOR to approximately the null value. Reliable ORs could not be estimated for epilepsy, as fewer than three cases were exposed to this maternal disorder. Due to small numbers of exposed cases for most variables in the three VACTERL subtypes, we were not able to estimate reliable ORs for the subtypes.

**TABLE 3:** Associations between maternal risk factors and the VACTERL association in offspring

	Registries included	Missing data	Total cases/controls	Cases exposed *N* (%)	Controls exposed *N* (%)	Crude OR (95% CI)	Adjusted OR (95% CI)
Multiple pregnancy	28	1.3%	328/49,098	17 (5.2)	1,380 (2.8)	1.9 (1.2–3.1)	0.6 (0.3–1.4)^a^
ART	16	15.0%	147/18,092	15 (10.2)	888 (4.9)	2.2 (1.3–3.8)	2.3 (1.3–3.9)^b^
Noninvasive	16	15.3%	146/18,027	7 (4.8)	342 (1.9)	2.7 (1.2–5.7)	2.8 (1.3–6.1)^b^
Invasive	16	15.3%	146/18,027	7 (4.8)	481 (2.7)	1.9 (0.9–4.1)	1.9 (0.9–4.0)^b^
Pregestational diabetes	15	22.6%	135/17,527	4 (3.0)	153 (0.9)	3.5 (1.3–9.5)	3.1 (1.1–8.6)^c^
CLOPD	14	23.8%	122/16,705	15 (12.3)	500 (3.0)	4.5 (2.6–7.9)	3.9 (2.2–6.7)^d^
Epilepsy	14	23.8%	122/16,705	2 (1.6)	84 (0.5)	‐	‐

*Note*: ORs were estimated if ≥3 cases were exposed. Registries with >50% missing data for a specific risk factor were excluded from the analyses for that factor.

Abbreviations: ART, assisted reproductive techniques; CI, confidence interval; CLOPD, chronic lower obstructive pulmonary disorders; OR, odds ratio.

a Adjusted for reporting registry, ART, and birth type.

b Adjusted for reporting registry.

c Adjusted for reporting registry, maternal age, and birth type.

d Adjusted for reporting registry and birth type.

In the sensitivity analyses using multiple imputation, similar risk estimates were obtained for all variables compared to the complete case analyses, except for the adjusted OR for multiple pregnancy (aOR 1.4 [95% CI 0.8, 2.4]) instead of aOR 0.6 (95% CI 0.3, 1.4) (eTable 1). As imputation did not change our interpretation of the results, we performed the remaining sensitivity analyses with the original data. In the sensitivity analyses with all VACTERL cases originally selected, including the NO‐VACTERL cases and the VACTERL cases with unknown subtype, similar risk estimates as those in Table 3 were obtained as well, albeit marginally lower ORs for pregestational diabetes and CLOPD (eTable 2). The maternal chronic illnesses were mainly identified based on the ICD‐9 and ICD‐10 codes for the specific diseases, but a small percentage of mothers were identified based on drug use only. When these mothers were included in the analyses, the ORs for pregestational diabetes were again marginally lower, whereas the ORs for CLOPD were slightly higher (eTable 3). After equalizing the proportions of TOPFAs among cases and controls by randomly excluding 15,894 controls, similar results were obtained as in the primary analysis (eTable 4). When we assumed that all mothers with unknown exposure to a specific risk factor were not exposed to that risk factor, we observed similar risk estimates as shown in Table 3 for multiple pregnancy and ART, whereas the risk estimates for pregestational diabetes and CLOPD were somewhat higher (eTable 5). Based on the ICD‐10‐BPA codes Q8730, Q8785, Q8717, and Q8715 or on less specific codes in combination with a specification in the text, 264 controls had imprinting disorders, while six controls had a diagnosis of caudal regression syndrome. After exclusion of these controls in the analyses for ART and pregestational diabetes, respectively, the risk estimates remained similar to those obtained in the primary analysis (eTable 6).

## 4 DISCUSSION

Among the maternal risk factors for VACTERL studied, we identified ART, pregestational diabetes, and CLOPD to be associated with VACTERL. No association was observed between multiple pregnancy and VACTERL after correction for ART and birth type. The number of cases exposed to epilepsy was too low to estimate reliable ORs.

An important strength of this study is that we performed the largest European case–control study to date to identify maternal risk factors for VACTERL using clear diagnostic criteria. Previously, we proposed three different VACTERL subtypes: STRICT‐VACTERL, VACTERL‐LIKE, and VACTERL‐PLUS, as well as a NO‐VACTERL subgroup (van de Putte, van Rooij, et al., 2019). Using this information, we were able to exclude the NO‐VACTERL cases from our study as they did not fulfill our diagnostic criteria for VACTERL. This most likely resulted in a more homogeneous case population compared to other studies with risk estimates that were less attenuated by nondifferential misclassification. This is illustrated by the sensitivity analysis in which we included the NO‐VACTERL cases (eTable 2) and showed risk estimates that were attenuated toward the null value. Using the remaining three VACTERL subtypes, we also tried to evaluate etiologic heterogeneity among these subtypes, but the sample sizes of exposed cases were too small to draw any meaningful conclusions.

Another strength is the inclusion of a range of sensitivity analyses, in which similar risk estimates were observed as in the primary analysis. Although some risk estimates were marginally higher or lower in specific analyses, they did not change the conclusions of our study.

A limitation is that no healthy control group was available, because EUROCAT only registers patients with congenital anomalies, genetic syndromes, or chromosomal abnormalities. A healthy control group is preferable, as these children do not have any congenital anomalies that may be caused by maternal risk factors. Previous studies showed, however, that a control group of patients with genetic syndromes and chromosomal abnormalities is comparable to the case group, because of similar recall errors (Bakker, de Walle, Dequito, van den Berg, & de Jong‐van den Berg, 2007; Lieff, Olshan, Werler, Savitz, & Mitchell, 1999). In addition, the exposure of such controls to maternal medication use and smoking was comparable to the general population of pregnant women (Bakker et al., 2007; Lieff et al., 1999). Nevertheless, some evidence indicates that maternal risk factors may be associated with genetic syndromes as well. Imprinting disorders, for example, were associated with ART (Manipalviratn et al., 2009) and caudal regression syndrome with pregestational diabetes (Garne et al., 2012). However, the numbers of patients with imprinting disorders or caudal regression syndrome were low in our control group, and the risk estimates did not change when controls with imprinting disorders and caudal regression syndrome were excluded from the specific analyses (eTable 6) As both the occurrence of several control disorders and multiple pregnancy, the use of ART, and chronic maternal illnesses increase with maternal age, we may not have been able to remove all confounding due to this factor. With the case mothers being younger on average, however, residual confounding by maternal age most likely resulted in underestimation of the effect estimates.

Another study limitation is the large number of missing data in some of the variables of interest, as these data were not collected by all registries. As collecting incomplete and inaccurate data are generally a waste of resources, it is justified for registries to only focus on data about the fetus/infant and its diagnosis. As the overall amount of missing data was extensive for body mass index and maternal education (>85%), we decided not to include these factors in this study. In addition, we excluded registries from specific analyses when data on the risk factor of interest were missing for >50% of the study population, as this reflects data that are not collected routinely, which could lead to serious selection bias. After exclusion of specific registries per risk factor studied, the proportions of missing data were 15–24%, except for maternal age, birth type, and multiple pregnancy (<2%). Multiple imputation of the missing data did not change our results, however.

Multiple pregnancy was not a risk factor for VACTERL in offspring in our study, after correction for ART and birth type. In contrast, associations with multiple pregnancy have been described for birth defects in general (Rider, Stevenson, Rinsky, & Feldkamp, 2013), and for ARM, including ARM with additional congenital anomalies or VACTERL (Wijers et al., 2013, 2014), nonsyndromic congenital heart defects (Ailes et al., 2014), and EA (Orford et al., 2000). However, most of these studies did not include ART in their multivariable models, whereas ART appeared to influence the risk estimate for multiple pregnancy greatly in this study.

ART is linked to an increased risk of congenital anomalies in general (Davies et al., 2012; Hansen, Bower, Milne, de Klerk, & Kurinczuk, 2005), but also of all individual components of VACTERL, along with other cardiovascular, urogenital, and musculoskeletal anomalies (Davies et al., 2012; Reefhuis et al., 2009; Wijers et al., 2013). The possibility of ART being involved in the etiology of VACTERL was already suggested by case reports (Kanasugi et al., 2013; Sunagawa et al., 2007). We showed that couples who conceived via ART had an increased risk of having a child with VACTERL. This association could be explained by hypocellularity due to the use of ART, which may also result in fetal growth restrictions (Lubinsky, 2017) and decreased birth weight (Dunietz et al., 2017). However, the question remains whether this can be attributed to the use of ART or to the underlying subfertility (Reefhuis et al., 2009; Wijers et al., 2015).

Prior to our study, the involvement of pregestational diabetes in the etiology of VACTERL had only been suggested by a few case reports (Castori et al., 2008) and one case only study (Garne et al., 2012). In this case–control study, we observed that women with pregestational diabetes had an increased risk of having a child with VACTERL. Pregestational diabetes, and specifically the corresponding hyperglycemia, has already been considered a human teratogen since the 1980s, when it became evident that it is associated with increased risks of isolated congenital anomalies affecting the central nervous system, the skeleton, the kidneys, the anorectal area, and the cardiovascular system (Garne et al., 2012; Liu et al., 2015; Mills, 1982; Wijers et al., 2014). The mechanism hypothesized for the involvement of pregestational diabetes in the etiology of congenital anomalies is an overload of glucose in embryonic mitochondria (Akazawa, 2005). Consequently, the production of reactive oxygen species (ROS) is increased, which may lead to an accumulation of mutations causing increased apoptosis resulting in congenital anomalies (Akazawa, 2005; Zabihi & Loeken, 2010). Therefore, it is important that women with pregestational diabetes achieve proper glycemic control in the periconceptional period. This was also suggested by Ray et al., who observed a lower prevalence of congenital anomalies among women who received extra preconception care, compared to women who did not receive care for optimization of glycemic control (Ray, O'Brien, & Chan, 2001).

An increased risk of VACTERL was also observed among mothers who had CLOPD, which were not linked to VACTERL before. Recently, a paper based on Dutch data was published on uncontrolled chronic respiratory diseases as a possible risk factor in the etiology of ARM, an important component of VACTERL (van de Putte, de Blaauw, et al., 2019). The hypothesis was posed that this risk factor is not specific for ARM, but may disturb embryologic development in general. Perhaps, this is also true for the etiology of VACTERL. Unfortunately, we were not able to make the distinction between controlled and uncontrolled respiratory diseases in the current study.

## 5 CONCLUSIONS

In the literature, several genetic risk factors in the etiology of the VACTERL association have been identified, but strong evidence for the involvement of maternal risk factors is lacking. We identified a role for several maternal risk factors in the etiology of VACTERL, specifically the use of ART, pregestational diabetes, and CLOPD. These risk factors have also been identified for several of the isolated components of VACTERL. This may indicate that the etiology of VACTERL is not very different from that of the isolated components. However, it does confirm that VACTERL is not solely caused by genetic risk factors, as we demonstrated a role for multiple maternal risk factors in the etiology of VACTERL, befitting a multifactorial etiology.

## CONFLICT OF INTEREST

The authors declare no conflict of interest.

## Supporting information


**eTable 1**. Associations between maternal risk factors and the VACTERL association in offspring after 50x multiple imputation
**eTable 2**. Associations between maternal risk factors and the VACTERL association in offspring including the NO‐VACTERL cases and the VACTERL cases with an unknown subtype
**eTable 3**. Associations between maternal chronic illnesses and the VACTERL association in offspring when maternal drug use was also taken into account to identify mothers with a chronic illness
**eTable 4**. Associations between maternal risk factors and the VACTERL association in offspring after equalizing the proportions of TOPFAs among cases and controls
**eTable 5**. Associations between maternal risk factors and the VACTERL association in offspring assuming that cases and controls with missing data on the determinants were not exposed
**eTable 6**. Associations between maternal risk factors and the VACTERL association in offspring after exclusion of controls with imprinting disorders and caudal regression syndrome

## Data Availability

The data that support the findings of this study are available from the EUROCAT registries. Restrictions apply to the availability of these data, which were used under license for this study. Data are available on request to JRC‐EUROCAT@ec.europa.eu, with the permission of the EUROCAT registries.
