# Supplementary material for: Maternal risk factors for the VACTERL association: A EUROCAT case–control study
Source: Birth Defects Res. 2020 Apr 22;112(9):688–98. doi: 10.1002/bdr2.1686 (PMC7319423; doi:10.1002/bdr2.1686)
Supplement: Supplementary file 1 — eTable 1. Associations between maternal risk factors and the VACTERL association in offspring after 50x multiple imputation eTable 2. Associations between maternal risk factors and the VACTERL association in offspring including the NO‐VACTERL cases and the VACTERL cases with an unknown subtype eTable 3. Associations between maternal chronic illnesses and the VACTERL association in offspring when maternal drug use was also taken into account to identify mothers with a chronic illness eTable 4. Associations between maternal risk factors and the VACTERL association in offspring after equalizing the proportions of TOPFAs among cases and controls eTable 5. Associations between maternal risk factors and the VACTERL association in offspring assuming that cases and controls with missing data on the determinants were not exposed eTable 6. Associations between maternal risk factors and the VACTERL association in offspring after exclusion of controls with imprinting disorders and caudal regression syndrome [file BDR2-112-688-s001.docx]

eTable 1. Associations between maternal risk factors and the VACTERL association in offspring after 50x multiple imputation.

|  | Registries  included | Missing  data | Total  cases/controls | Cases exposed  N (%) | Controls exposed  N (%) | Crude OR  (95% CI) | Adjusted OR  (95% CI) |
| --- | --- | --- | --- | --- | --- | --- | --- |
| Multiple pregnancy | 28 | 0% | 329/49724 | 17 (5.2) | 1,400 (2.8) | 1.9 (1.2, 3.1) | 1.4 (0.8, 2.4)^b^ |
| ART | 16 | 0% | 163/21,288 | 17 (10.4) | 1,060 (5.0) | 2.3 (1.3, 3.9) | 2.3 (1.4, 4.0)^c^ |
| Non-invasive^a^ | 16 | 15.3% | 146/18,027 | 7 (4.8) | 342 (1.9) | 2.7 (1.2, 5.7) | 2.8 (1.3, 6.1)^c^ |
| Invasive^a^ | 16 | 15.3% | 146/18,027 | 7 (4.8) | 481 (2.7) | 1.9 (0.9, 4.1) | 1.9 (0.9, 4.0)^c^ |
| Pregestational diabetes | 15 | 0% | 151/22,655 | 4 (2.6) | 214 (0.9) | 3.1 (1.1, 8.4) | 2.9 (1.0, 7.9)^d^ |
| CLOPD | 14 | 0% | 139/21,931 | 18 (12.9) | 697 (3.2) | 4.4 (2.6, 7.6) | 3.8 (2.2, 6.5)^e^ |
| Epilepsy | 14 | 0% | 139/21,931 | 2 (1.4) | 122 (0.6) | **-** | **-** |

ART = assisted reproductive techniques, CLOPD = chronic lower obstructive pulmonary disorders, OR = odds ratio, CI = confidence interval. ORs were estimated if ≥3 cases were exposed. Registries with >50% missing data for a specific risk factor were excluded from the analyses for that factor.

^a^ For ART invasiveness, imputation was not performed as this information was dependent on ART use.

^b^ Adjusted for reporting registry, ART, and birth type.

^c^ Adjusted for reporting registry.

^d^ Adjusted for reporting registry, maternal age, and birth type.

^e^ Adjusted for reporting registry and birth type.

eTable 2. Associations between maternal risk factors and the VACTERL association in offspring including the NO-VACTERL cases and the VACTERL cases with an unknown subtype.

|  | Registries  included | Missing  data | Total  cases/controls | Cases exposed  N (%) | Controls exposed  N (%) | Crude OR  (95% CI) | Adjusted OR  (95% CI) |
| --- | --- | --- | --- | --- | --- | --- | --- |
| Multiple pregnancy | 28 | 1.3% | 417/49,098 | 26 (6.2) | 1,380 (2.8) | 2.3 (1.5, 3.4) | 0.9 (0.4, 1.7)^a^ |
| ART | 16 | 15.0% | 168/18,092 | 17 (10.1) | 888 (4.9) | 2.2 (1.3, 3.6) | 2.2 (1.3, 3.7)^b^ |
| Non-invasive | 16 | 15.3% | 167/18,027 | 7 (4.2) | 342 (1.9) | 2.3 (1.1, 5.0) | 2.5 (1.1, 5.3)^b^ |
| Invasive | 16 | 15.3% | 167/18,027 | 9 (5.4) | 481 (2.7) | 2.1 (1.1, 4.2) | 2.1 (1.1, 4.2)^b^ |
| Pregestational diabetes | 15 | 22.5% | 160/17,527 | 4 (2.5) | 153 (0.9) | 2.9 (1.1, 8.0) | 2.6 (0.9, 7.1)^c^ |
| CLOPD | 14 | 23.7% | 147/16,705 | 17 (11.6) | 500 (3.0) | 4.2 (2.5, 7.1) | 3.5 (2.1, 6.0)^d^ |
| Epilepsy | 14 | 23.7% | 147/16,705 | 2 (1.4) | 84 (0.5) | - | - |

ART = assisted reproductive techniques, CLOPD = chronic lower obstructive pulmonary disorders, OR = odds ratio, CI = confidence interval.

ORs were estimated if ≥3 cases were exposed. Registries with >50% missing data for a specific risk factor were excluded from the analyses for that factor.

^a^ Adjusted for reporting registry, ART, and birth type.

^b^ Adjusted for reporting registry.

^c^ Adjusted for reporting registry, maternal age, and birth type.

^d^ Adjusted for reporting registry and birth type.

eTable 3. Associations between maternal chronic illnesses and the VACTERL association in offspring when maternal drug use was also taken into account to identify mothers with a chronic illness.

|  | Registries  included | Missing  data | Total  cases/controls | Cases exposed  N (%) | Controls exposed  N (%) | Crude OR  (95% CI) | Adjusted OR  (95% CI) |
| --- | --- | --- | --- | --- | --- | --- | --- |
| Pregestational diabetes | 15 | 22.6% | 135/17,525 | 4 (3.0) | 170 (1.0) | 3.1 (1.1, 8.5) | 2.8 (1.0, 7.8)^a^ |
| CLOPD | 14 | 23.7% | 123/16,709 | 18 (14.6) | 541 (3.2) | 5.1 (3.1, 8.5) | 4.4 (2.6, 7.3)^b^ |
| Epilepsy | 14 | 23.7% | 122/16,711 | 2 (1.6) | 105 (0.6) | **-** | **-** |

CLOPD = chronic lower obstructive pulmonary disorders, OR = odds ratio, CI = confidence interval.

ORs were estimated if ≥3 cases were exposed. Registries with >50% missing data for a specific risk factor were excluded from the analyses for that factor.

^a^ Adjusted for reporting registry, maternal age, and birth type.

^b^ Adjusted for reporting registry and birth type.

eTable 4. Associations between maternal risk factors and the VACTERL association in offspring after equalizing the proportions of TOPFAs among cases and controls.

|  | Registries  included | Missing  data | Total  cases/controls | Cases exposed  N (%) | Controls exposed  N (%) |  | Crude OR  (95% CI) | Adjusted OR  (95% CI) |
| --- | --- | --- | --- | --- | --- | --- | --- | --- |
| Multiple pregnancy | 28 | 1.6% | 328/33,285 | 17 (5.2) | 1079 (3.2) |  | 1.6 (1.0, 2.7) | 0.6 (0.3, 1.5)^a^ |
| ART | 16 | 15.8% | 147/11,615 | 15 (10.2) | 561 (4.8) |  | 2.2 (1.3, 3.8) | 2.3 (1.3, 4.0)^b^ |
| Non-invasive | 16 | 16.1% | 146/11,574 | 7 (4.8) | 219 (1.9) |  | 2.7 (1.2, 5.8) | 2.8 (1.3, 6.2)^b^ |
| Invasive | 16 | 16.1% | 146/11,574 | 7 (4.8) | 301 (2.6) |  | 1.9 (0.9, 4.2) | 2.0 (0.9, 6.2)^b^ |
| Pregestational diabetes | 15 | 22.4% | 135/11,460 | 4 (3.0) | 120 (1.0) |  | 2.9 (1.1, 7.9) | 3.3 (1.2, 9.2)^c^ |
| CLOPD | 14 | 23.5% | 122/10,994 | 15 (12.3) | 372 (3.4) |  | 4.0 (2.3, 6.9) | 4.0 (2.3, 6.9)^b^ |
| Epilepsy | 14 | 23.5% | 122/10,994 | 2 (1.6) | 57 (0.5) |  | **-** | **-** |

TOPFA = terminations of pregnancy for fetal anomaly following prenatal diagnosis, ART = assisted reproductive techniques, CLOPD = chronic lower obstructive pulmonary disorders, OR = odds ratio, CI = confidence interval. ORs were estimated if ≥3 cases were exposed. Registries with >50% missing data for a specific risk factor were excluded from the analyses for that factor.

^a^ Adjusted for reporting registry and ART.

^b^ Adjusted for reporting registry.

^c^ Adjusted for reporting registry and maternal age.

eTable 5. Associations between maternal risk factors and the VACTERL association in offspring assuming that cases and controls with missing data on the determinants were not exposed.

|  | Registries  included | Missing  data | Total  cases/controls | Cases exposed  N (%) | Controls exposed  N (%) | Crude OR  (95% CI) | Adjusted OR  (95% CI) |
| --- | --- | --- | --- | --- | --- | --- | --- |
| Multiple pregnancy | 28 | 0% | 329/49,724 | 17 (5.2) | 1,380 (2.8) | 1.9 (1.2, 3.1) | 0.6 (0.3, 1.4)^a^ |
| ART | 16 | 0% | 163/21,288 | 15 (9.2) | 888 (4.2) | 2.3 (1.4, 4.0) | 2.4 (1.4, 4.1)^b^ |
| Non-invasive | 16 | 0.3% | 162/21,223 | 7 (4.3) | 342 (1.6) | 2.8 (1.3, 6.1) | 3.0 (1.4, 6.5)^c^ |
| Invasive | 16 | 0.3% | 162/21,223 | 7 (4.3) | 481 (2.3) | 2.0 (0.9, 4.3) | 2.0 (0.9, 4.3)^c^ |
| Pregestational diabetes | 15 | 0% | 151/22,655 | 4 (2.6) | 153 (0.7) | 4.0 (1.5, 10.9) | 3.7 (1.4, 10.3)^d^ |
| CLOPD | 14 | 0% | 139/21,931 | 15 (10.8) | 500 (2.3) | 5.2 (3.0, 8.9) | 4.5 (2.6, 7.7)^e^ |
| Epilepsy | 14 | 0% | 139/21,931 | 2 (1.4) | 84 (0.4) | **-** | **-** |

ART = assisted reproductive techniques, CLOPD = chronic lower obstructive pulmonary disorders, OR = odds ratio, CI = confidence interval. ORs were estimated if ≥3 cases were exposed. Registries with >50% missing data for a specific risk factor were excluded from the analyses for that factor.

^a^ Adjusted for reporting registry, ART, and birth type.

^b^ Adjusted for reporting registry.

^c^ Adjusted for reporting registry. A small percentage of missing data is still present as the type of ART was unknown.

^d^ Adjusted for reporting registry, maternal age, and birth type.

^e^ Adjusted for reporting registry and birth type.

eTable 6. Associations between maternal risk factors and the VACTERL association in offspring after exclusion of controls with imprinting disorders and caudal regression syndrome.

|  | Registries  included | Missing  data | Total  cases/controls | Cases exposed  N (%) | Controls exposed  N (%) | Crude OR  (95% CI) | Adjusted OR  (95% CI) |
| --- | --- | --- | --- | --- | --- | --- | --- |
| ART | 16 | 14.9% | 147/17,828 | 15 (10.2) | 867 (4.9) | 2.2 (1.3, 3.8) | 2.3 (1.3, 3.9)^a^ |
| Non-invasive | 16 | 15.2% | 146/17,764 | 7 (4.8) | 335 (1.9) | 2.7 (1.3, 5.8) | 2.9 (1.3, 6.2)^a^ |
| Invasive | 16 | 15.2% | 146/17,764 | 7 (4.8) | 468 (2.6) | 1.9 (0.9, 4.1) | 1.9 (0.9, 4.1)^a^ |
| Pregestational diabetes | 15 | 22.6% | 135/17,521 | 4 (3.0) | 152 (0.9) | 3.5 (1.3, 9.6) | 3.1 (1.1, 8.6)^b^ |

ART = assisted reproductive techniques, CLOPD = chronic lower obstructive pulmonary disorders, OR = odds ratio, CI = confidence interval.

ORs were estimated if ≥3 cases were exposed. Registries with >50% missing data for a specific risk factor were excluded from the analyses for that factor.

^a^ Adjusted for reporting registry. The analyses were performed after exclusion of controls with the following imprinting disorders: Beckwith-Wiedemann syndrome, Angelman syndrome, Silver-Russell syndrome, and Prader-Willi syndrome.

^b^ Adjusted for reporting registry, maternal age, and birth type. The analyses were performed after exclusion of controls with caudal regression syndrome.
